# Magnetic resonance imaging based 3-dimensional printed breast surgical guide for breast-conserving surgery in ductal carcinoma in situ: a clinical trial

**DOI:** 10.1038/s41598-020-75398-7

**Published:** 2020-10-28

**Authors:** Zhen-Yu Wu, Aisha Alzuhair, Heejeong Kim, Jong Won Lee, Il Yong Chung, Jisun Kim, Sae Byul Lee, Byung Ho Son, Gyungyub Gong, Hak Hee Kim, Joon Beom Seo, Sei Hyun Ahn, Namkug Kim, BeomSeok Ko

**Affiliations:** 1 Division of Breast Surgery, Department of Surgery, Asan Medical Center, University of Ulsan College of Medicine, 88, Olympic-ro 43-gil, Songpa-gu, Seoul, 05505 Republic of Korea; 2 Department of Breast Surgery, Shanghai East Hospital, Tongji University School of Medicine, Shanghai, China; 3 Department of Surgery, College of Medicine, King Faisal University, Al Hofuf, Saudi Arabia; 4 Department of Pathology, Asan Medical Center, University of Ulsan College of Medicine, 88, Olympic-ro 43-gil, Songpa-gu, Seoul, 05505 Republic of Korea; 5 Department of Radiology, Asan Medical Center, University of Ulsan College of Medicine, 88, Olympic-ro 43-gil, Songpa-gu, Seoul, 05505 Republic of Korea

**Keywords:** Breast cancer, Surgical oncology, Clinical trials

## Abstract

Breast-conserving surgery (BCS) is performed in patients with ductal carcinoma in situ (DCIS) because of the small size of the tumor. It is essential to know the quantitative extent of the tumor before performing this precise partial resection surgery. A three-dimensional printed (3DP) breast surgical guide (BSG) was developed using information obtained from supine magnetic resonance imaging (MRI) and 3D printing technology and it was used for treating patients with breast cancer. Here, we report our experience with the application of the BSG for patients with DCIS. Patients with breast cancer who underwent BCS from July 2017 to February 2019 were included in this study. The patients underwent partial resection with a supine-MRI based 3DP-BSG. A total of 102 BCS using 3DP-BSG were conducted, and 11 cases were DCIS. The patients’ median age was 56 years (range, 38–69 years). The mean tumor diameter was 1.3 ± 0.9 cm. The median surgical time was 70 min (range, 40–88 min). All patients had tumor-free resection margins. The median distance from the tumor to the margin was 11 mm (range, 2–35 mm). Direct demarcation of the tumor extent in the breast and a pain-free procedure are the advantages of using 3DP-BSG in patients with DCIS.

Trial registration: Clinical Research Information Service (CRIS) Identifier Number: KCT0002375, KCT0003043.

## Introduction

The incidence of ductal carcinoma in situ (DCIS) has increased over time because of the implementation of active screening examinations for breast cancer^1,2^. Partial resection is generally attempted for treating small DCIS, but in some cases, the tumor involves a larger area than expected based on preoperative breast imaging or it is not easily palpable. The frequency of tumor-positive margins is reportedly 13–58%^3^, which is closely related to the re-excision and recurrence rates. It is essential to accurately determine the extent of the tumor to be included in the partial resection in order to achieve a clean margin.

Magnetic resonance imaging (MRI) is more accurate than other imaging methods such as mammography and ultrasonography. Several localization methods have been used to obtain improved surgical results in breast-conserving surgery (BCS), among which wire-guided localization (WGL) is the most commonly used. Although the procedure is relatively simple, its disadvantages include vasovagal reactions, and cutting, moving, or loss of the wire^4–6^. Radio-guided occult lesion localization (ROLL) involves injecting a liquid radioactive tracer (^99m^Tc) around the lesion a few hours before the tumor resection surgery^7,8^. Gray et al. described a procedure for radioactive seed localization (RSL) for BCS^9^. RSL provides better cosmetic results with lower rates of positive margins and re-excision than WGL. However, some studies did not find any difference between WGL and RSL^10,11^. Radiation exposure caused by the use of radioactive substances and problems with managing the seeds are drawbacks of RSL.

However, the chief disadvantage of all of the existing methods of localization is that they are not compatible with MRI-guided localization, which predicts the extent of DCIS most accurately. Therefore, we created a three-dimension printed (3DP)-breast surgical guide (BSG) using data obtained from supine MRI combined with 3D printing technology and used it for performing BCS in patients with DCIS.

## Methods

### Eligibility

This study was approved by the Institutional Review Board of Asan Medical Center (No. 2016-1237, 2018-0690) and it was performed in accordance with the principles of the Declaration of Helsinki. All patients provided voluntary informed consent to participate in the study. Women with a confirmed diagnosis of breast cancer from July 2017 to February 2019, who were aged 18 to 69 years and were available for radiological and physical evaluations after BCS were eligible for enrollment in this prospective single-center cohort study. We excluded men and patients with contraindications for MRI. Patients provided written informed consent and agreed to undergo supine imaging in addition to the standard baseline MRI protocol.

### Production of the surgical guide

**Figure 1:**
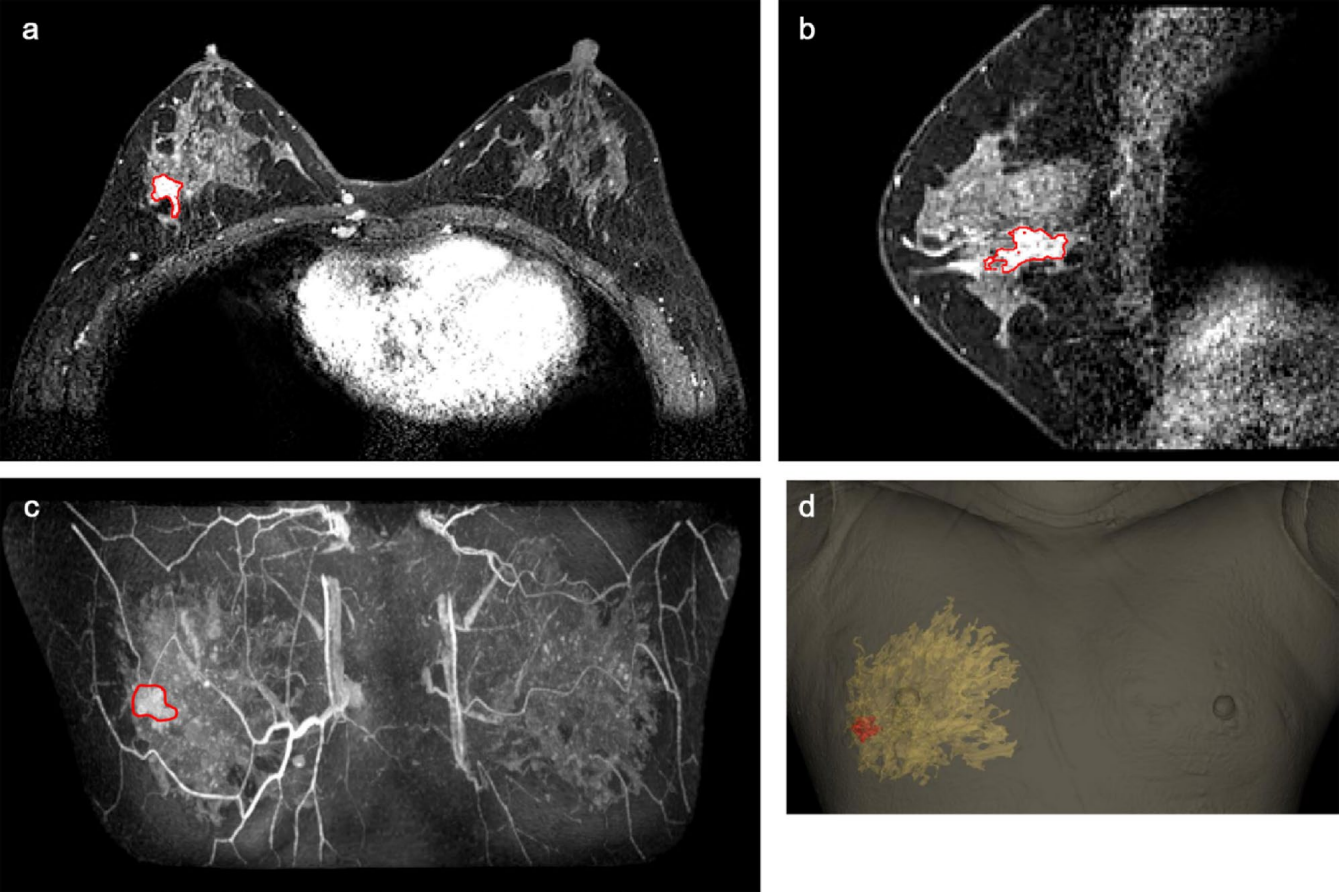
Segmentation of normal tissues and tumors in breast MRI. (**A**, **B**) Tumor segmentation in prone MRI. (**C**) Tumor segmentation in supine MRI. (**D**) 3D modeled breast and tumor based on prone/supine MRI.

**Figure 2:**
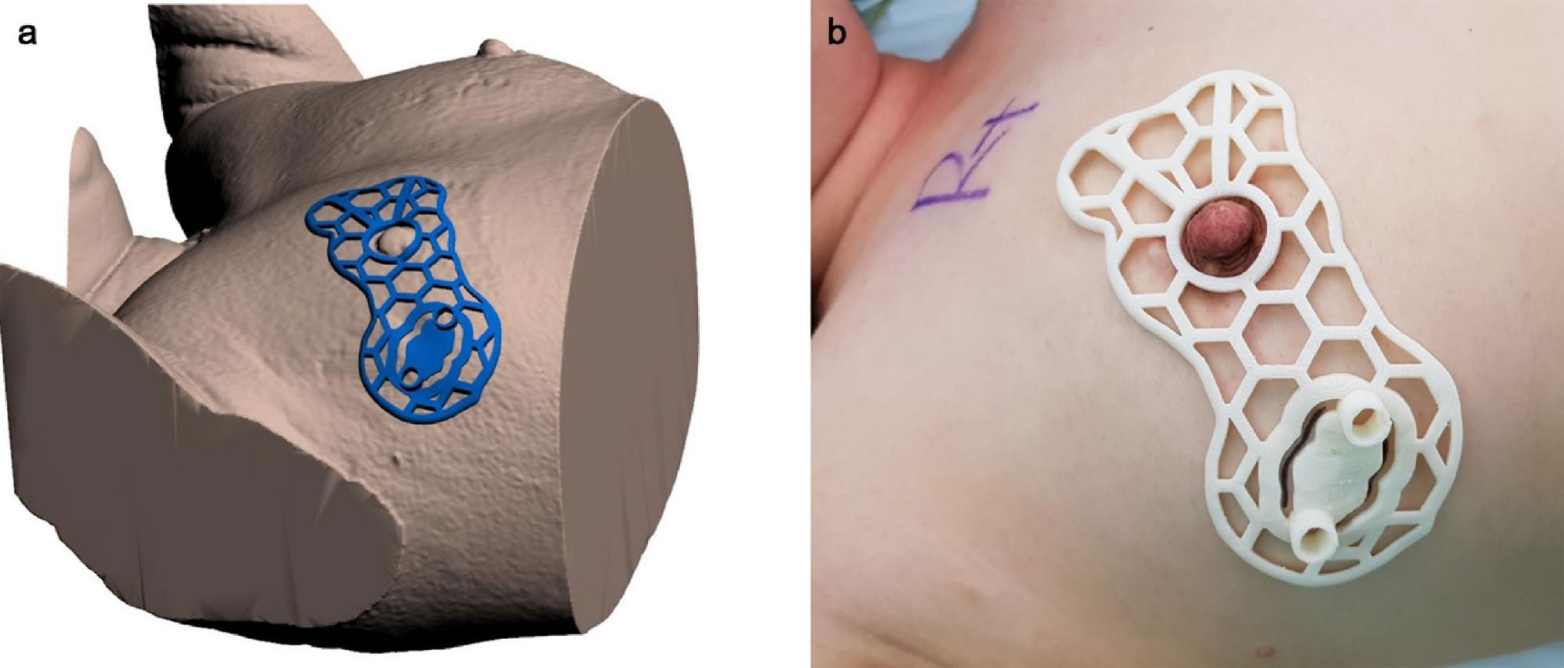
Production of BSGs and its application on the patients. (**A**) BSG modeling to target tumors using 3D patient images. BSG is designed to have columns that can mark the tumor boundary inside the breast by injecting a blue dye and a groove for skin surface marking. (**B**) Patient-tailored BSG designed for tumor targeting applied during surgery.

Breast imaging was performed using a 3.0 T MRI system (Ingrain; Philips Healthcare, Netherlands) with a bilateral dedicated four-element breast coil. Both arms were raised above the head, and additional MRI was performed with the patient in the supine position to replicate the patient’s position during the surgical procedure. Data obtained from the prone/supine MRI scans were analyzed, and the tumors and normal tissues were divided using the image segmentation program Mimics Medical 17 (Materialise Inc., Belgium) (Fig. 1A–D). The BSG was modeled at a distance of 0.5 cm from the tumor boundary to guarantee a margin of safety. The following specifications were used for modeling the BSG to ensure an accurate display of the tumor resection boundary: (1) it was made to fit precisely to the breast skin surface, (2) a hole was provided to fit the nipple, and (3) guidelines to prevent rotation of the BSG and to indicate its placement relative to the opposite nipple and the suprasternal notch were included. The BSGs were made to fit on the patient's skin surface and were manufactured in a hybrid type with a groove for marking the breast surface and a column for precisely marking the underlying tissue (Fig. 2A, B).

### Surgical and pathologic assessment

**Figure 3:**
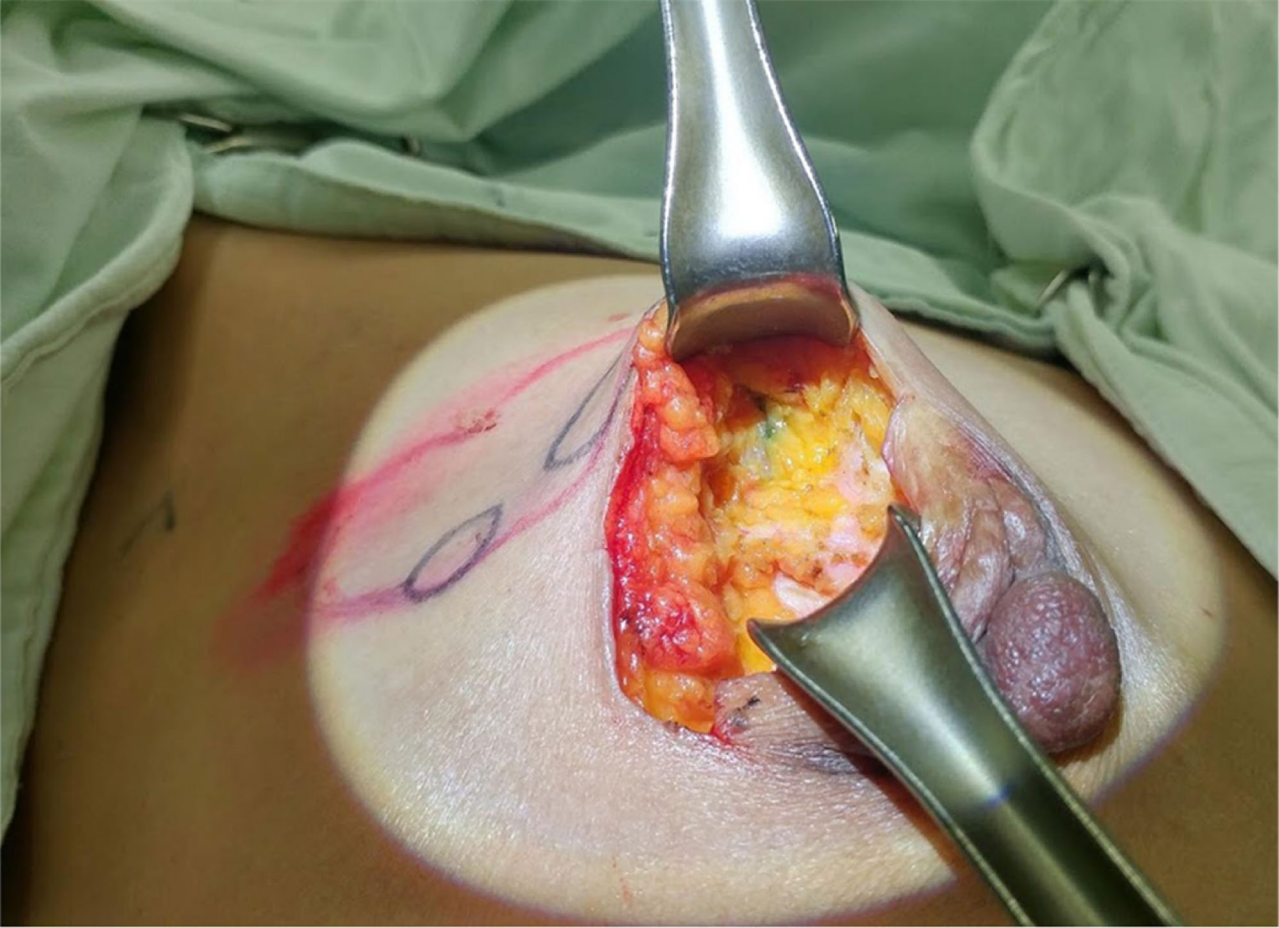
Blue dye injected around the tumor using BSG.

Patient-specific BSGs were printed and sterilized preoperatively. The BSG was fixed to both nipples, and the suprasternal notch was used as a landmark. The tumor resection boundary was drawn along the line designed to match the tumor shape. Blue dye was injected through the column to indicate the extent to be removed around the tumor. The breast tissue was removed using the blue border as the excision boundary (Fig. 3). Some tissue samples were obtained from several cavities to confirm the status of the margins intraoperatively after partial resection using frozen biopsy. Resection was continued until a tumor-free margin was obtained for tumor-positive samples. Mastectomy was performed if tumor-positive margins were observed at several sites and preservation of the breast tissue was judged to be difficult. The absence of continuity of the breast tissue was not considered to have any effect on recurrence; thus, it was not included in the evaluation of the resected margins^12^. Sentinel node biopsy was performed for large tumors.

Pathological evaluation was performed after removing the entire tumor bed and the state of the margin was confirmed. The distance from the tumor edge to the resection margin was measured at the 3, 6, 9, and 12 o'clock positions.

## Results

BCS was performed with the 3DP-BSG in 11 patients with DCIS during the study period. The patient and tumor characteristics are shown in Table 1. The median age of the patients was 56 years (range 38–69 years). Four patients had multiple tumors, including invasive breast cancer, but only the DCIS lesions were analyzed. DCIS was diagnosed by core needle biopsy before surgery in 8 patients, but the lesions were not observed by mammography or ultrasonography in 3 patients, only by MRI. DCIS was confirmed during the final pathological examination in all included cases.

**Table 1:** Patient and tumor characteristics.

Variables		N (%)
Age, years	Median	56
	Range	38–69
	≤ 50	4 (36.4)
	> 50	7 (63.6)
Microinvasion	Yes	3 (27.3)
	No	8 (72.7)
Tumor size, cm	≤ 2	7 (63.6)
	> 2	4 (36.4)
Lymph node status	Negative	10 (90.9)
	Positive	1 (9.1)
Nuclear grade	I	1 (9.1)
	II	7 (63.6)
	III	3 (27.3)
Multifocality	Yes	5 (45.5)
	No	6 (54.6)
Subtype	HR+/HER2−	8 (72.7)
	HR+/HER2+	1 (9.1)
	HR−/HER2+	2 (18.2)
	TN	0 (0)
Axillary surgery	SLNB	11 (100)
	ALND	0 (0)

*SLNB* sentinel lymph node biopsy, *ALND* axillary lymph node dissection, *HR* hormone receptor, *HER2* human epidermal growth factor receptor 2, *TN* triple negative.

The surgical specimen characteristics are shown in Table 2. The mean tumor diameter was 1.3 ± 0.9 cm. The median surgical time was 70 min (range 40–88 min); the surgical time was longer for patients with multiple lesions. Sentinel node biopsy was performed in all patients. Pathological examination revealed tumor-free resection margins in all patients and that the median size of the DCIS lesions was 6 mm (range 3–25 mm). The median distance from the tumor to the margin was 11 mm (range 2–35 mm). Nine patients with positive hormone receptors were treated with antihormone therapy as an adjuvant to surgery. Chemotherapy was performed in 1 patient with multiple invasive tumors, and targeted therapy was administered in 1 patient. All patients underwent radiotherapy.

**Table 2:** Surgical specimen characteristics.

Variables		N (%)
Margin status, frozen	Negative	9 (81.8)
	Positive	2 (18.2)
Margin status, permanent	Negative	11 (100)
	Positive	0 (0)
Operation time, min	Mean ± SD	67 ± 17
	Median	70
	Range	40–88
Nearest margin, cm	Mean ± SD	0.8 ± 0.4
	Median	0.9
	Range	0.2–1.5
Tumor diameter, cm	Mean ± SD	1.3 ± 0.9
	Median	0.6
	Range	0.3–2.5
Specimen diameter, cm	Mean ± SD	5.2 ± 1.6
	Median	5.0
	Range	3.8–9.5
Tumor-to-margin distance, cm	Mean ± SD	1.2 ± 0.7
	Median	1.1
	Range	0.2–3.5

*SD* standard deviation

## Discussion

Several methods have been devised to localize DCIS since it usually manifests as a non-palpable tumor. WGL is the most commonly used localization method and it is relatively easy to perform under mammography or ultrasonography-guidance. However, it is difficult to determine the overall extent of the tumor using this method. In 1987, Silverstein et al. proposed a method of inserting multiple wires around the tumor. Targeting the wrong area during the procedure, hematoma, pneumothorax and complications such as cutting, migration, and loss of the wires during surgery are the disadvantages of WGL^4–6^.

RSL was first reported by Gray et al.^9^ The procedure involves inserting a radioactive iodine (^125^I) seed into the tumor under the guidance of mammography or ultrasonography before surgery, and using a gamma detector probe to remove the surrounding tumor tissue. Several studies reported low rates of tumor-positive margins and re-excisions, and a lower volume of breast tissue resection with RSL than that with WGL^9,13^, along with better cosmetic results. However, ROLL and RSL both require strict management and are associated with the risk of radiation exposure. Besides, Rampaul RS et al. found no significant difference in the accuracy of localization, mean duration of surgery, specimen weight, and need for intraoperative re-excision or second therapeutic surgery with RSL as compared with ROLL and WGL.

Sharek et al.^10^ compared RSL and WGL in 232 cases; they failed to find significant differences in the surgical margin, re-excision rate, reoperation rate, ratio of the tumor volume to initial surgical specimen volume, ratio of the tumor volume to total volume resected, or in the final clinical cosmesis scores. Peter et al.^14^ reported that there were no differences in positive margins rates, positive or close margins rates, specimen volume, weight, reoperation, or operation time in 153 cases in the WGL group and 152 cases in the RSL group.

Various imaging tests are performed to identify the extent of the tumor accurately before surgery. The accuracy of MRI for detecting DCIS is higher than that of mammography or ultrasonography, and MRI is often performed when DCIS is diagnosed. However, it is difficult to directly mark the area of the tumor on the MRI scan using conventional methods. Saka et al. reported the use of supine MRI to draw a lesion on the breast using the projection technique during DCIS resection. However, this method cannot always accurately target tumors inside the breast. 3DP-BSG can use MRI data to directly mark the extent of the tumor on the skin and provide a quantitative representation of the extent of the tumor (by injecting blue dye through columns that are modeled in three-dimensions) to target the tumor within the breast.

In 3 cases in this study, the tumor was not seen on ultrasonography, and it was observed as indeterminate small nodules only by MRI, and thus a preoperative biopsy was not performed. BSG was applied and the lesions were removed. The final pathological result was diagnosed as DCIS. DCIS might have recurred in these patients if instead of 3DP-BSG, ultrasonography-guided tumor resection was performed according to conventional methods.

Since the 3DP-BSG is pre-fabricated, it makes the surgical procedure less time-consuming, and it does not cause pain because it is only applied after general anesthesia. There is no risk of migration, loss, or radiation exposure. Above all, its advantage over conventional localization techniques is that precise surgery is made possible by quantitatively marking the tumor area directly on the breast based on MRI.

The main limitations of the current study were the small sample size and the lack of a cosmetic results analysis after surgery. However, using MRI-based 3DP-BSG, we believe that precise targeting can completely remove tumors while preserving the normal tissues as much as possible, leading to good cosmetic results.
